# Special Issue: Bacteriophage Treatment as an Alternative Technology to Inactivate Pathogenic Bacteria: A Generalized Worldwide Growing Acceptance

**DOI:** 10.3390/microorganisms10010012

**Published:** 2021-12-23

**Authors:** Adelaide Almeida

**Affiliations:** Departamento de Biologia e CESAM, Universidade de Aveiro, 3810-193 Aveiro, Portugal; aalmeida@ua.pt

The increasing worldwide rate of antibiotic resistance has led to a higher incidence of bacterial infections that require alternative methods for their control not only in human medicine, but also in other areas, such as in veterinary medicine, agro-food field and wastewater treatment.

Phage therapy has emerged as an effective solution against antibiotic-resistant bacterial strains that can be used as a substitute or adjuvant to antibiotic therapy. Although in Eastern Europe phage therapy traditions and practices began in 1919 and continue today in a clinical setting, in Western Europe and the USA phage therapy continues to lack any market approval, but has been increasingly used as an experimental therapy for the compassionate treatment of patients experiencing antibiotic failure [1,2,3]. Nonetheless, soon after the appearance of the first successful results of phage therapy in human medicine, the relevant knowledge was quickly translated to other areas such as veterinary medicine, the food industry, and agriculture and aquaculture, where this approach has been well received and some applications have already been approved [4,5,6,7,8,9].

Nevertheless, while the efficacy of phage therapy has proven it to be an efficient alternative to conventional antibiotics, there is still need for new developments to translate the approach into routine treatments. Some important applications of phage therapy in the clinical field that require urgent development, besides fighting infections caused by multidrug-resistant bacteria, are the treatment of infections caused by intracellular bacteria, infections caused by bacteria capable of forming biofilms, bacterial chronic infections, and even the treatment of bacterial systemic infections. In non-clinical fields, it is imperative to study the impact of phage use in the environment as well as the effect of abiotic factors on phage viability—such as temperature, pH, salinity and UV radiation—particularly if phage treatment will be used outside, where these parameters vary greatly throughout the year. The structural and functional stabilization/preservation of phage particles in supports may be an important strategy to overcome the negative effect of these abiotic factors. Another important aspect, for both clinical and non-clinical applications, is the need to prevent potential bacterial regrowth after treatment due to the development of phage-resistant mutants, which can be hampered by the use of phage cocktails (which at the same time can broaden the action spectrum of the phages) and/or by the use of combined approaches, such as the use of antibiotics during phage treatment. Additionally, in general, more ex vivo and in vivo studies are also imperative to translate the technology to the field.

This Special Issue highlights recent advances in some of these areas. It includes fourteen papers focused on the use of phage therapy in a clinical setting, namely, to fight infections caused by multidrug-resistant bacteria [10,11,12] and by bacteria capable of forming biofilms [13], and in non-clinical fields, namely in the agro-food sector [14,15,16,17], including also the inactivation of bacteria forming biofilms [18,19]. Two of the papers [11,17] focus on the development of phage-resistant mutants after phage therapy, which is an important aspect for both clinical and non-clinical applications. Five of the published papers in this Special Issue refer to some strategies to prevent the development of phage-resistant mutants, namely by the use of combined approaches, such as the use of antibiotics [12,20], fatty acids [18], essential oil [19], and mucin [21] during phage treatment. Four of the papers include in vivo studies in animals [14,15,22] and plants [23].

As regards the use of phage therapy in a clinical setting to fight infections caused by multidrug-resistant bacteria, the results of the papers included in this Special Issue showed that lytic phages belonging to different families—the *Siphoviridae* family [10] and *Podoviridae* family [11]—have the potential for controlling infections caused by multidrug-resistant *Klebsiella pneumoniae*, and that a mutated lysogenic phage displayed antimicrobial activity against multidrug-resistant *Acinetobacter baumannii* [12]—two important common pathogenic bacteria in human medicine.

Since one of the main virulence factors of pathogenic bacteria is their ability to form biofilm structures, which commonly occurs in clinical multidrug-resistant bacteria and is not controlled by antibiotics, the use of phages is an alternative approach for the eradication of bacterial biofilms. In the study by Fiscarelli [13], five newly isolated environmental phages showed wide host activity against *Pseudomonas aeruginosa* strains capable of forming biofilms isolated from patients with cystic fibrosis [13].

As regards the use of phage therapy in non-clinical fields, the papers included in this Special Issue showed that phages are effective for fighting infections in aquaculture and agriculture. For the aquaculture field, in the study by Donati et al. [14] the potential of bacteriophages against the pathogenic bacteria *Flavobacterium psychrophilum* and *F. columnare* on rainbow trout eyed eggs was evaluated. The results demonstrated a strong potential for short term (24 h) phage control of *F. psychrophilum*. Moreover, it was found that phages do not negatively affect the survival of rainbow trout eyed eggs and do not strongly adhere to the surface of eyed eggs. Kim et al. [15] also evaluate the effectiveness of phage therapy in aquaculture for inactivating *Vibrio coralliilyticus*, one of the major pathogens causing mass mortality in marine bivalve larvae aquaculture. They isolated two *Podoviridae* bacteriophages that specifically infected pathogenic *V. coralliilyticus* from an oyster hatchery. Both phages were stable over a wide range of temperatures (4–37 °C) and at pH 7.0–9.0, being suitable for application under the environmental conditions of an oyster hatchery. The two phages showed confirmed significant bactericidal efficacy against pathogenic *V. coralliilyticu* in vitro. In vivo, the phage pre-treated groups of Pacific oyster larvae showed significantly lower mortality against *V. coralliilyticus* infection than untreated control larvae, suggesting that both phages could be used in the artificial marine bivalve seedling industry to prevent pathogenic *V. coralliilyticus* infection [15].

In agriculture studies, phage therapy was tested against *Pectobacterium odoriferum*, a bacterium that has recently emerged as a widely infective and destructive pathogen causing soft-rot disease in various vegetables, such as in Kimchi cabbage [16]. A new isolated bacteriophage showed lytic activity against *P. odoriferum* and two other *Pectobacterium* species. The phage significantly inhibited the development of soft-rot disease in the mature leaves of harvested Kimchi cabbage up to 48 h after inoculation compared to the untreated leaves, suggesting that phages can protect Kimchi cabbage from soft-rot disease after harvest, thereby presenting prophylactic and therapeutic potential in the control of bacterial soft-rot disease [16]. Pinheiro et al. [17] also described a phage application in the agriculture field. They evaluated the efficacy of bacteriophage φ6 (a commercially available phage) for controlling *Pseudomonas syringae* pv. *Syringae*, the main causative agent of diseases in a wide variety of fruit trees. The results showed that the phage infects not only its host (*P. syringae* pv. *syringae*), but also two strains of *Pseudomonas syringae* pv. *Actinidiae*, the causal agent of bacterial canker of kiwifruit. The viability of phage φ6 was mostly affected by exposure to UV-B irradiation and to solar radiation, and by high temperatures, but not affected by temperatures of 15 °C and 25 °C. The results suggest that this phage can be used to control *P. syringae* pv. *Syringae* infections in plants, but also infections by *P. syringae* pv. *actinidiae*. Although the stability of phage φ6 was affected by UV-B and solar radiation, this can be overcome by the application of phage suspensions at the end of the day or at night.

Although anti-biofilm approaches with phages in environmental microbiology are still rare, in this Special Issue two agriculture biofilm studies were included. In the agriculture area, phage therapy combined with fatty acids showed effective activity against mature biofilms of *Xanthomonas campestris* pv. *campestris*, the causative agent of black rot disease, which attacks mainly crucifers and severely lowers their global productivity [18]. Also, phage therapy combined with an essential oil, the carvacrol, presented activity against *Pseudomonas syringae* pv. *Actinidiae* (Psa), the causative agent of the bacterial canker of kiwifruit [19]. The phages and the carvacrol alone prevented biofilm formation, but the combined treatment prevented Psa regrowth and also destroyed the pre-formed biofilms [19].

Although phage therapy is a promising approach for controlling the emergence and spread of multidrug-resistant bacteria, the rapid development of anti-phage resistance has been identified as an obstacle to the development of phage therapy in both clinical and non-clinical applications. However, Pinheiro et al. [17] showed low rates of development of phage-resistant bacterial clones (1.20 × 10^−3^), suggesting that resistance cannot limit the use of phage therapy for controlling bacterial infection by *Pseudomonas syringae*. The study by Tan et al. [11] studied the mechanism employed by multidrug-resistant *K. pneumoniae* strains and the manner in which they protect themselves from lytic phage predation in vitro and in vivo. They performed comparative genomic analysis, and showed that undecaprenyl-phosphate glucose-1-phosphate transferase (WcaJ), the initial enzyme catalyzing the biosynthesis of colanic acid, is necessary for the adsorption of the phage studied, phage 117 (a *Podoviridae*), to the host strain, strain Kp36, in order to complete its lytic life cycle. In-frame deletion of *wcaJ* alone was sufficient to provide phage 117 resistance in the Kp36 wild-type strain. They also demonstrated that the susceptibility of phage 117 and the mucoid phenotype could be restored in the resistant strain Kp36-117R by expressing the wild-type version of *wcaJ*. They found that bacterial mobile genetic elements (*insA* and *insB*) block phage 117 infections by disrupting the coding region of *wcaJ*, preventing phage adsorption to its phage receptor. They also showed that the *wcaJ* mutation likely occurred spontaneously rather than as an adaptation to phage 117 predation under unfavorable environments. Their results address a crucial evolutionary question around the mechanisms of phage–host interactions, increasing the current understanding of anti-phage defense mechanisms in important multidrug-resistant pathogens [11].

Some strategies have been established for preventing the development of phage-resistant mutants by the use of combined approaches, including antibiotics [12,20], fatty acids [18], essential oil [19], and mucin [21], which were addressed in this Special Issue.

The combination of phages and antibiotics was evaluated in two studies. Davis et al. [20] tested the correct combination of phages and antibiotics to produce synergistic inhibitory effects on *Pseudomonas aeruginosa*. In this study, they tested sub-MIC levels of the antibiotic aztreonam lysine (AzLys) and phages E79 and phiKZ, specific to *P. aeruginosa*. Phage E79 plus AzLys PAS was able to significantly reduce *P. aeruginosa* biofilm growth over 3-fold as compared to the phage treatment alone. Phage phiKZ also produced phage-antibiotic synergistic killing with sub-inhibitory AzLys. In contrast with prior phage-antibiotic synergy studies demonstrating that phages undergo delayed time to lysis with cell filamentation. These phage-antibiotic synergy results show that phages undergo accelerated time to lysis, which therefore suggests that phage-antibiotic synergy is dependent upon multiple factors, including the type of phages and antibiotics used and the bacterial host being tested [20]. Blasco et al. [12] used the mutated lysogenic phage that displayed antimicrobial activity against an *A. baumannii* clinical strain with a meropenem and imipenem minimum inihibitory concentration (MIC) of, respectively, 32 µg/mL and 16 µg/mL. They observed an in vitro synergistic antimicrobial effect (reduction of 4 log–7 log CFU/mL) between meropenem and the lytic phage in all combinations analyzed (meropenem at 1/4 and 1/8 MIC). Moreover, bacterial growth was reduced by 8 log CFU/mL for the combination of imipenem at 1/4 MIC plus lytic phage and by 4 log CFU/mL for the combination of imipenem at 1/8 MIC plus lytic phage. These results were confirmed in an in vivo model (*Galleria mellonella*), and the combination of phage and imipenem was the most effective. The authors concluded that this approach could help to reduce the emergence of phage resistant bacteria and restore sensitivity to antibiotics used to combat multi-resistant strains of *Acinetobacter baumannii*.

The combination of phages and fatty acids was also evaluated by Papaianni et al. [18] for the *Xanthomonas campestris* pv. *Campestris*, as mentioned before, the causative agent of black rot disease, which has the capability to penetrate and form biofilm structures in the xylem vessels. Considering the multifactorial nature of biofilm, an effective approach against *Xanthomonas campestris* implies the use of a multi-targeted or combinatorial strategy. In this study, the authors tested the use of fatty acids and the bacteriophage (Xccφ1)–hydroxyapatite complex against *Xanthomonas campestris* mature biofilm. The synergic action of the combined therapy was efficient for removing *Xanthomonas campestris* mature biofilm, showing that the approach is an effective solution to enhance plant survival during *Xanthomonas campestris* infections. Ni et al. [19] also tested the combined effect of a phage with an essential oil, the carvacrol, against *Pseudomonas syringae* pv. *Actinidiae* (Psa), which, as mentioned before, is the causative agent of the bacterial canker of kiwifruit. The combination of two phages (single phage suspensions of phages PN05 and PN09, and a cocktail of both phages) and carvacrol was investigated in controlling Psa planktonic and biofilm forms in vitro. The phage therapy alone (with phages PN05 and PN09), and the carvacrol alone (minimum inhibitory concentration 2.0 mg/mL), inhibited Psa growth, but the combined effect of both therapies was more effective. The phages alone effectively inhibited Psa growth for 24 h, but Psa regrowth was observed after this time. The carvacrol (2.0 mg/mL) alone prevented biofilm formation for 48 h, but did not destroy the pre-formed biofilms. The combined treatment, with both phages and carvacrol (2.0 mg/mL), showed a higher efficacy, preventing Psa regrowth for more than 40 h. The authors concluded that the combined treatment with phages and carvacrol may be a promising, environmentally friendly and cost-effective approach to controlling Psa in the kiwifruit industry. Carrol-Portilo & Lin [21] reviewed the effect of mucin combined with phages as a therapy for gastrointestinal (GI) dysfunction. The authors concluded that the community-level structural changes in the gut microbiota may require an alternative to conventional phage therapy, such as a phage cocktail capable of targeting multiple bacterial species. They also stated that the manipulation of the GI microenvironment may enhance beneficial bacteria-phage interactions during treatment. Mucin, produced along the entire length of the GI tract to protect the underlying mucosa, is a prominent contributor to the GI microenvironment and may facilitate bacteria-phage interactions in multiple ways, potentially serving as an adjunct during phage therapy for gastrointestinal dysbiosis.

Several studies have reported on the isolation and characterization of new phages and their efficiency in controlling pathogenic bacteria. However, few in vivo assays have been reported so far. One of the challenges that phage therapy studies face is to demonstrate its feasibility in in vivo and in field conditions [24,25,26], because in vitro assays are not enough to understand phage-bacteria interactions that occur in vivo, which makes difficult the translation of the technology to the field. In this Special Issue, three of the papers include in vivo studies in animals [14,15] and plants [23], showing effective bacterial inactivation, confirming the potential of phage therapy to be used in the field. However, in vivo assays are not always feasible, even when model organisms are used. A fourth paper from this Special Issue, by Nale et al. [22], treats of refining the *Galleria mellonella* model by using stress marker genes to assess *Clostridioides difficile* infection and recuperation during phage therapy. *G. mellonella* has been considered an effective model for probing bacterial interactions with phages, but despite valuable insights from this model, the larvae are not easily amenable to assessing detailed clinical responses to either bacteria or phages. In this study, larval survival, colonisation and toxin levels were compared to expression profiles of 17 *G. mellonella* stress genes to monitor *Clostridiodes difficile* infection and recuperation during phage therapy. Larvae treated prophylactically with phages and the phage-control larval group were protected, showing the highest survival and low *C. difficile* colonisation and toxin rates compared to co-infection, remedial and bacterial-control larval groups. Expression of growth (9) and reproduction (2) genes was enhanced within prophylaxis and phage-control larval groups compared to the co-infection, remedial and bacterial control groups. In contrast, expression of infection (2), humoral (1) and cellular (3) immunity genes declined in the prophylactic and phage-control groups but increased in the co-infection, remedial and bacterial control larvae. The molecular markers augment the survival, colonisation and toxin data and allow detailed monitoring of *Clostridiodes difficile* infection and recovery. This data support the use of stress marker genes as tools to analyse clinical symptoms in this model [22], which can help to translate the phage therapy technology to the field.

Altogether, these fourteen papers published in this Special Issue gathered the most recent knowledge from researchers demonstrating that bacteriophages are promising candidates for controlling bacterial infection not only in the clinical field, but also in other areas, such as in aquaculture and agriculture. In consideration of the many aspects related to optimal phage therapy in the inactivation of bacteria, it was my pleasure to edit a joint presentation of the results from different research groups in one special scientific publication challenging researchers to respond to its current underutilization in clinical and environmental applications.

## References

[1] Górski A., Borysowski J., Międzybrodzki R. (2020). Phage Therapy: Towards a Successful Clinical Trial. Antibiotics.

[2] McCallin S., Sacher J.C., Zheng J., Chan B.K. (2019). Current State of Compassionate Phage Therapy. Viruses.

[3] Luong T., Salabarria A.-C., Roach D.R. (2020). Phage Therapy in the Resistance Era: Where Do We Stand and Where Are We Going?. Clin. Ther..

[4] Wojtasik A., Górecka E., Wójcik E., Stańczyk M., Kołsut J., Klimczak J., Dastych J., Siwicki A., Schulz P. (2017). Bacteriophage Strains and Their. Applications. Patent.

[5] Fernández L., Gutiérrez D., Rodríguez A., García P. (2018). Application of bacteriophages in the agro-food sector: A long way toward approval. Front. Cell Infect. Microbiol..

[6] Pereira C., Costa P., Pinheiro L., Balcão V.M., Almeida A. (2021). Kiwifruit bacterial canker: An integrative view focused on biocontrol strategies. Planta.

[7] Pereira C., Costa P., Duarte J., Balcão V.M., Almeida A. (2021). Phage therapy as a potential approach in the biocontrol of pathogenic bacteria associated with shellfish consumption. Int. J. Food Microbiol..

[8] Loponte R., Pagnini U., Iovane G., Pisanelli G. (2021). Phage Therapy in Veterinary Medicine. Antibiotics.

[9] Ferriol-González C., Domingo-Calap P. (2021). Phage Therapy in Livestock and Companion Animals. Antibiotics.

[10] Peng Q., Fang M., Liu X., Zhang C., Liu Y., Yuan Y. (2020). Isolation and Characterization of a Novel Phage for Controlling Multidrug-Resistant Klebsiella pneumoniae. Microorganisms.

[11] Tan D., Zhang Y., Qin J., Le S., Gu J., Chen L., Guo X., Zhu T. (2020). A Frameshift Mutation in wcaJ Associated with Phage Resistance in Klebsiella pneumoniae. Microorganisms.

[12] Blasco L., Ambroa A., Lopez M., Fernandez-Garcia L., Bleriot I., Trastoy R., Ramos-Vivas J., Coenye T., Fernandez-Cuenca F., Vila J. (2019). Combined Use of the Ab105-2φΔCI Lytic Mutant Phage and Different Antibiotics in Clinical Isolates of Multi-Resistant Acinetobacter baumannii. Microorganisms.

[13] Fiscarelli E.V., Rossitto M., Rosati P., Essa N., Crocetta V., Di Giulio A., Lupetti V., Di Bonaventura G., Pompilio A. (2021). In Vitro Newly Isolated Environmental Phage Activity against Biofilms Preformed by Pseudomonas aeruginosa from Patients with Cystic Fibrosis. Microorganisms.

[14] Donati V.L., Dalsgaard I., Runtuvuori-Salmela A., Kunttu H., Jørgensen J., Castillo D., Sundberg L.-R., Middelboe M., Madsen L. (2021). Interactions between Rainbow Trout Eyed Eggs and Flavobacterium spp. Using a Bath Challenge Model: Preliminary Evaluation of Bacteriophages as Pathogen Control Agents. Microorganisms.

[15] Kim H.J., Giri S.S., Kim S.W.G., Kim S.W.G., Kwon J., Lee S.B., Park S.C. (2020). Isolation and Characterization of Two Bacteriophages and Their Preventive Effects against Pathogenic Vibrio coralliilyticus Causing Mortality of Pacific Oyster (Crassostrea gigas) Larvae. Microorganisms.

[16] Lee S., Vu N.-T., Oh E.-J., Rahimi-Midani A., Thi T.-N., Song Y.-R., Hwang I.-S., Choi T.-J., Oh C.-S. (2021). Biocontrol of Soft Rot Caused by Pectobacterium odoriferum with Bacteriophage phiPccP-1 in Kimchi Cabbage. Microorganisms.

[17] Pinheiro L.A.M., Pereira C., Frazão C., Balcão V.M.V., Almeida A. (2019). Efficiency of Phage φ6 for Biocontrol of Pseudomonas syringae pv. syringae: An in Vitro Preliminary Study. Microorganisms.

[18] Papaianni M., Ricciardelli A., Casillo A., Corsaro M.M., Borbone F., Della Ventura B., Velotta R., Fulgione A., Woo S.L., Tutino M.L. (2020). The Union Is Strength: The Synergic Action of Long Fatty Acids and a Bacteriophage against Xanthomonas campestris Biofilm. Microorganisms.

[19] Ni P., Wang L., Deng B., Jiu S., Ma C., Zhang C., Almeida A., Wang D., Xu W., Wang S. (2020). Combined Application of Bacteriophages and Carvacrol in the Control of Pseudomonas syringae pv. actinidiae Planktonic and Biofilm Forms. Microorganisms.

[20] Davis C.M., McCutcheon J.G., Dennis J.J. (2021). Aztreonam Lysine Increases the Activity of Phages E79 and phiKZ against Pseudomonas aeruginosa PA01. Microorganisms.

[21] Carroll-Portillo A., Lin H.C. (2021). Exploring Mucin as Adjunct to Phage Therapy. Microorganisms.

[22] Nale J.Y., Chutia M., Cheng J.K.J., Clokie M.R.J. (2020). Refining the Galleria mellonella Model by Using Stress Marker Genes to Assess Clostridioides difficile Infection and Recuperation during Phage Therapy. Microorganisms.

[23] Flores O., Retamales J., Núñez M., León M., Salinas P., Besoain X., Yañez C., Bastías R. (2020). Characterization of Bacteriophages against Pseudomonas Syringae pv. Actinidiae with Potential Use as Natural Antimicrobials in Kiwifruit Plants. Microorganisms.

[24] Park S.C., Nakai T. (2003). Bacteriophage control of Pseudomonas plecoglossicida infection in ayu Plecoglossus altivelis. Dis. Aquat. Organ..

[25] Silva Y.J., Costa L., Pereira C., Mateus C., Cunha Â., Calado R., Gomes N.C.M.C.M., Pardo M.A.A., Hernandez I., Almeida A. (2014). Phage therapy as an approach to prevent Vibrio anguillarum infections in fish larvae production. PLoS ONE.

[26] Silva Y., Moreirinha C., Pereira C., Costa L., Rocha R.J.M., Cunha Â., Gomes N.C.M., Calado R., Almeida A. (2016). Biological control of Aeromonas salmonicida infection in juvenile Senegalese sole (Solea senegalensis) with Phage AS-A. Aquaculture.

